# Solid and Semisolid Innovative Formulations Containing Miconazole-Loaded Solid Lipid Microparticles to Promote Drug Entrapment into the Buccal Mucosa

**DOI:** 10.3390/pharmaceutics13091361

**Published:** 2021-08-29

**Authors:** Viviana De Caro, Libero Italo Giannola, Giulia Di Prima

**Affiliations:** Dipartimento di Scienze e Tecnologie Biologiche Chimiche e Farmaceutiche (STEBICEF), University of Palermo, Via Archirafi 32, 90123 Palermo, Italy; litagian@gmail.com (L.I.G.); giulia.diprima@unipa.it (G.D.P.)

**Keywords:** miconazole, solid lipid microparticles, penetration enhancer, oral candidiasis, buccal gel, buccal film, cetyl decanoate, ex vivo studies, mucosal delivery, buccal mucosa

## Abstract

The currently available antifungal therapy for oral candidiasis (OC) has various limitations restricting its clinical use, such as short retention time, suboptimal drug concentration and low patients compliance. These issues could be overcome using micro or nanotechnology. In particular, solid lipid microparticles (SLMs) resulted as a particularly promising penetration enhancer carrier for lipophilic drugs, such as the antifungal miconazole (MCZ). Based on these considerations, cetyl decanoate (here synthesized without the use of metal catalysis) was employed together with 1-hexadecanol to prepare MCZ-loaded SLMs. These resulted in a powder composed of 45–300 µm diameter solid spherical particles, able to load a high amount of MCZ in the amorphous form and characterized by a melting temperature range perfectly compatible with oromucosal administration (35–37 °C). Moreover, when compared to Daktarin^®^ 2% oral gel in ex vivo experiments, SLMs were able to increase up to three-fold MCZ accumulation into the porcine buccal mucosa. The prepared SLMs were then loaded into a buccal gel or a microcomposite mucoadhesive buccal film and evaluated in terms of MCZ permeation and/or accumulation into porcine buccal mucosa by using lower doses than the conventional dosage form. The promising results obtained highlighted an enhancement in terms of MCZ accumulation even at low doses. Furthermore, the prepared buccal film was eligible as stable, reproducible and also highly mucoadhesive. Therefore, the formulated SLMs represent a penetration enhancer vehicle suitable to reduce the dose of lipophilic drugs to be administered to achieve the desired therapeutic effects, as well as being able to be effectively embedded into easily administrable solid or semisolid dosage forms.

## 1. Introduction

Oral candidiasis (OC) is a common oral fungal disease generally caused by Candida albicans. Even if Candida albicans is a physiological component of the oral cavity’s microflora when an imbalance occurs, it can cause infectious disease [1,2,3]. In particular, OC is a superficial disorder that affects the oral mucous membranes, and it is generally characterized by removable white plaques and generalized redness of the tissue or erosive erythroplakia and leukoplakic lesions, or chronic, discrete plaque-like or nodular lesions, depending on the severity of the pathology. Furthermore, associated lesions (e.g., denture stomatitis) could be involved in the pathophysiology of the OC. Due to the wide damage of the mucosal surface, patients often suffer from dysgeusia, burning, tenderness and dysphagia. Nowadays, the accepted treatment for oral candidiasis is the use of topical antifungal agents, such as miconazole [4,5,6]. Miconazole (1-[2-(2,4-dichlorobenzyloxy)-2-(2,4-dichlorophenyl)ethyl]imidazole; C_18_H_14_Cl_4_N_2_O; logP: 5.3) is a synthetic imidazole antifungal that has been used for nearly 40 years to treat superficial fungal infections effectively and safely. Miconazole (MCZ) acts by damaging the integrity of the fungal cell membrane, altering fungal adherence and inhibiting the formation of germ tubes and mycelia. MCZ has potent wide-spectrum activity against many Candida species. Furthermore, it is also useful and effective against several species of Candida that are resistant to fluconazole [7,8]. Anyhow, the currently available topical treatment has various disadvantages and limitations restricting its clinical use. In particular, the short retention time on the oral mucosa results in the need for multiple applications each day, makes it difficult to reach effective drug concentration and thus optimal effects and limits the treatment compliance. Moreover, the elderly generally find the formulations technically difficult to apply to all the locations of the oral cavity, while young people also require an effective and easy-to-apply treatment [7,9]. In recent years, innovative dosage forms have been designed for buccal drug delivery in order to overcome the main limitations of this administration route. Conventional dosage forms are generally liquid, semisolid (e.g., gels and pastes), or solid dosage forms (e.g., capsules and tablets). Each novel formulation aims to improve drug retention on the application site, ease of administration and thus treatment effectiveness and compliance [9,10]. The use of micro and nanotechnology should result in an innovative strategy to promote drug effectiveness by enhancing its penetration and accumulation into the mucosal tissue. In particular, solid lipid microparticles (SLMs) consist of a hydrophobic core, solid at room temperature, able to embed the active ingredient and dissolve or disperse into the solid lipid matrix. They are mainly composed of physiologically compatible and biodegradable constituents, providing well in vivo tolerability and optimal biodegradability. SLMs are generally characterized by good physicochemical stability, high loading capacity for lipophilic and poorly water-soluble molecules, ability to regulate drug release rate and increase its bioavailability. SLMs can also serve to protect drugs from degradation. The production of SLMs is also feasible at a large scale, avoiding the use of organic solvents, with a low production cost [11,12,13]. Furthermore, their similarity with the lipid constituents of the mucosal tissue could result in improved interaction with the surface of application, enhanced drug retention and accumulation into the tissue and, consequently, increased drug effectiveness. In addition, SLMs exhibit some interesting advantages compared to solid lipid nanoparticles (SLNs). The micrometric dimensions (from 20 to 300 µm) of SLMs actually permit the obtainment of a solid free-flowing powder, stable, easy to handle and workable, which could represent an intermediate for the preparation of various solid and semisolid pharmaceutical dosage forms. Moreover, for specific administration routes (e.g., nasal and pulmonary) or applications (e.g., skin and mucosal delivery), their micrometric size is the most appropriate. A wide variety of lipophilic excipients can be used to prepare SLMs. The choice of the suitable lipid carrier is of crucial importance when preparing these drug delivery systems, as the selected excipients will affect the properties of the whole preparation [13].

Based on these considerations, the aims of the present work are the following: propose an innovative and useful strategy to synthetized cetyl decanoate without the use of metal catalysis, employ the obtained pure cetyl decanoate to prepare MCZ-loaded SLMs with ad hoc properties (e.g., suitable melting point for buccal application) and finally embed MCZ-loaded SLMs into semisolid or solid formulations (gel or microcomposite thin film, respectively) in order to make them easily dosable and administrable.

## 2. Materials and Methods

### 2.1. Materials

Miconazole (MCZ, CAS 22916-47-8) and citric acid were purchased from Farmalabor (Bari, Italy). Decanoic acid chloride, 1-hexadecanol, Polyvinylpyrrolidone K90 (PVP-K90) and (R)-(+)-limonene were purchased from Sigma Aldrich (Steinheim, Germany).

Hydroxyethylcellulose (HEC, Natrosol™ 250 MR) was supplied by Galeno (Firenze, Italy). Buffer solution (pH 6.8) simulating saliva was prepared by dissolving KCl (1.50 g), KSCN (0.54 g), NaH_2_PO_4_·H_2_O (0.50 g), NaHCO_3_ (1.50 g) and lactic acid (0.90 g) in 1 L of distilled water.

Phosphate Buffer Saline solution pH 7.4 (PBS) was prepared by dissolving 2.80 g of KH_2_PO_4_ and 20.5 g of Na_2_HPO_4_ in 1 L of distilled water. All these components were purchased from VWR International (Leuven, Belgium). Trehalose was supplied by Hayashibara Shoij (Hayashibara Shoij Inc., Okayama, Japan).

All solvents and chemicals were of analytical grade and were used without further purification. Porcine mucosae specimens were kindly supplied by the Municipal Slaughterhouse of Villabate (Palermo, Italy).

### 2.2. Methods

#### 2.2.1. Synthesis and Characterization of Cetyl Decanoate

Decanoic acid chloride (95.36 g, 0.5 mol) was dissolved in anhydrous dichloromethane (50 mL), and the clear solution was carefully added to a previously heated (60 ± 1 °C) 1-hexadecanol (121.22 g, 0.5 mol) solution in anhydrous dichloromethane (50 mL) under continuous stirring. The reaction was kept at 60 ± 1 °C under reflux for 12 h. During the reaction, the HCl produced was eliminated by evaporation, and the precipitation of a product was observed. At the end of the reaction, the mixture was evaporated off under reduced pressure, and the crude product was washed several times in NaHCO_3_ saturate solution and finally in distilled water until pH 7 was reached. The solid waxy mass obtained was filtered, completely dried under vacuum and repeatedly crystallized from acetone and ethanol until gas-chromatographic purity (>98%). Yield: >80%. Four recrystallizations were sufficient to produce analytically pure samples of ester.

The characterization of the obtained product comprises NMR and FTIR analysis, as well as the determination of the melting point. Spectral data were in line with the expected structure.

^1^H NMR and ^13^C NMR spectra were recorded using a Bruker Avance II 300 spectrometer operating at 300.12 MHz. for ^1^H and 75.47 MHz for ^13^C equipped with a 5-mm ^1^H/^13^C dual probe.

^1^H NMR (300 MHz, CDCl_3_): δ = 0.88 (br t, 6 H), 1.26 (br s, 38 H), 1.58–1.64 (br m, 4 H), 2.27–2.35(t, J = 7.5 Hz, 2 H), 4.04–4.12 (t, J = 6.7 Hz, 2 H).

^13^C NMR (75 MHz, CDCl_3_): δ = 14.15, 22.68, 25.03, 25.94, 28.66, 29.16, 29.28, 29.42, 29.55, 29.69, 29.84, 31.91, 34.40, 64.38, 173.99.

FTIR spectra were recorded on a Fourier Transform Infrared Spectrometer (FTIR) (Spectrum Two FTIR spectrometer, Perkin Elmer, Waltham, MA, USA) ranging from 4000 to 450 cm^−1^ as nujol mulls. The spectra were obtained as the mean of 20 scans. IR (cm^−1^): 1737 and 1473 (ester C=O).

The melting points (m.p.) were determined by a Büchi B-540 capillary apparatus (heating rate: 0.5 °C/min) and are uncorrected.

#### 2.2.2. Screening of 1-Hexadecanol and Cetyl Decanoate Mixtures

Different ratios of 1-hexadecanol and cetyl decanoate mixtures (from 10:90 to 90:10 *w*/*w*), including MCZ (10% *w*/*w*) were prepared and evaluated in terms of melting point and morphology of the obtained SLMs (see below) in order to highlight the best composition.

#### 2.2.3. Preparation of Miconazole (MCZ) Loaded Solid Lipid Microparticles (SLMs)

To prepare the SLMs, the hot-melt technique was employed. Miconazole (10% *w*/*w* of the lipid mixture), 1-hexadecanol and cetyl decanoate (at various ratios) were melted using a heating plate (Heidolph MR 10 3001 K, Heidolph Instrument GmbH & CO., Schwabach, Germany) equipped with a thermostatic probe (Heidolph EKT 3001, Heidolph Instrument GmbH & CO., Schwabach, Germany). Afterward, 100 mL of previously heated water (90 °C) containing NaHCO_3_ (0.5 g) and limonene (1 mL) were added under continuous stirring (800 rpm) using a Polimix mechanical stirrer RW20, equipped with a digital speed indicator KCH-TRON (Kinematica, Malters, Switzerland) and a steel 4-blade rod (diameter 4.5 cm) in order to obtain an O/W emulsion. After 2 min of constant stirring, the prepared emulsion was cooled by immersing the beaker into an ice bath, and the continuous stirring was kept until the temperature reached 10 ± 2 °C. The cooling procedure leads to the solidification of the oil phase, thus allowing the obtaining of the SLMs. The prepared SLMs were then filtered and dried at room temperature. For further studies, the chosen best formulation was the following: MCZ:1-hexadecanol:cetyl decanoate = 10:72:18 ratio. Yield 97.0 ± 0.5%, referring to the starting amount of the total lipid and drug mass employed.

#### 2.2.4. Drug Loading (DL%) and Loading Efficacy (LE%)

A total of 5 mg of SLMs was transferred into a 5 mL volume flask, completely dissolved in methanol and brought to volume with fresh methanol. The amount of MCZ was determined spectrophotometrically (UV-VIS Shimadzu 1700 Instrument, Kyoto, Japan) at λ = 272 nm. MCZ standard solutions were used to construct the calibration curve (concentration range: 0.05–0.50 mg/mL; y = 1.821x + 0.041; R = 0.995). The second derivative technique was used to exclude any potential interference of the excipients employed. The DL% (drug loading percentage) was calculated as the amount of MCZ (mg) encapsulated into 100 mg of SLMs. The LE% (loading efficacy percentage) was calculated as the amount of MCZ (mg) actually encapsulated into the SLMs relating to the total amount of MCZ (mg) used. The quantification of MCZ in each SLMs batch was performed in triplicate.

#### 2.2.5. Morphological Analysis of SLMs

To evaluate surface morphology and particle geometric characteristics, optical microscopy analysis was conducted. To collect the photographs of some representative samples of SLMs, a Leica M165C (Leica Microsystems, Wetzlar, Germany) optical microscope equipped with a Photometrics digital camera DFC450 and Leica application Suite LAS4 software (40× magnification) was used.

#### 2.2.6. Particle Size Evaluation

- By Light Scattering analysis:

The aqueous dispersion of SLMs (0.1 mg/mL) was subjected to particle size distribution analysis by light scattering. Measurements were carried out at 25 ± 0.5 °C by using a Mastersizer 3000 instrument (Malvern Panalytical Ltd., Malvern, UK) fitted with both a blue (470 nm) and a red (632.8 nm) laser at a fixed scattering angle of 144°. Each experiment was performed in triplicate.

- By sieving analysis:

The separation of the microparticles into various size fractions was carried out by using an Endecotts Octagon 200 test sieve shaker (Endecotts Ltd., London, UK) and standard mesh wire sieves (Endecotts). A series of seven standard stainless-steel sieves (range 45–310 µm) were arranged in the order of decreasing aperture size. Each batch (about 2 g) was placed on the upper sieve of the series. The sieves were mounted on the mechanical shaker operating for 15 min continuously. The average particles size of each fraction was determined as the arithmetic mean of the aperture size of the screen they were retained upon and the aperture size of the screen that they passed. The weight of separated materials was measured, and the size distribution was determined. Data were reported as mean ± SE of six batches.

#### 2.2.7. Determination of SLM Melting Temperature Range

Each batch of SLMs was inserted into a capillary glass, and the melting temperature range was investigated by a Büchi B-540 capillary apparatus (BÜCHI Labortechnik AG, Essen, Germany) carrying out a heating rate of 0.5 °C/min until the microparticles entirely melted. Each sample was evaluated in triplicate.

#### 2.2.8. Differential Scanning Calorimetry (DSC)

DSC analyses were performed by using a Perkin-Elmer Pyris Diamond (Perkin-Elmer, Waltham, MA, USA). Samples (about 8–10 mg) were hermetically sealed in Perkin-Elmer aluminum caps with the aid of a punching machine and then tested from −30 to 130 °C (heating rate of 10 °C/min). Indium was used as the calibration standard. Each analysis was performed in triplicate.

#### 2.2.9. Preparation of SLMs-Loaded Buccal Gel

Trehalose (150 mg), PVP-K90 (50 g) and Hydroxyethylcellulose (HEC—800 mg) were added to 50 mL of previously heated distilled water (60 °C), gently stirred until complete dissolution of each component and then left at room temperature for 24 h, until a homogeneous gel was obtained. Afterward, SLMs (ratio SLMs:gel = 1:8 *w*/*w*) were added and manually incorporated into the previously prepared gel in order to obtain a homogeneous and air bubbles-free dispersion.

The uniformity of MCZ content was assessed by UV-Vis analysis. Exactly weighted samples of 100 mg of gel (*n* = 6) were withdrawn from each batch of formulation and dissolved into a distilled water/methanol mixture (1:4 *v*/*v*) and then brought to volume (5 mL) with the same solvents mixture.

The amount of MCZ was determined at λ = 272 nm. MCZ standard solutions in distilled water/methanol (1:4 *v*/*v*) were used to construct the calibration curve (concentration range: 0.05–0.50 mg/mL; y = 1.689x + 0.00317; R = 0.999).

#### 2.2.10. Preparation and Characterization of SLMs-Loaded Buccal Thin Films

##### Preparation of Hydrophilic (Empty) Buccal Thin Films

Thin layered films, as hydrophilic matrix intended to incorporate the SLMs, were prepared by the solvent casting method starting from aqueous gels. Four different gel compositions (Table 1) were tested in order to identify the best one in terms of thinness, flexibility and transparency.

Trehalose (when present) was dissolved in pre-warmed distilled water (15 mL, 45 °C), and then PVP-K90, HEC and another 15 mL of water were added. Finally, limonene was added (when present). The obtained gels were left in an ultrasonic bath (Branson B1200, Branson Ultrasonic Corporation, Danbury, CT, USA) for 2 h in order to promote polymers swelling and mixing, as well as to remove any air bubble. Afterward, 5 g of each gel were placed into silicone molds (4.5 × 4.5 cm; area 20.25 cm^2^) and dried in an oven (StabiliTherm, Thermo Fisher Scientific) at 30 °C and 55% of humidity (analogical thermohygrometer VWR International, Milan, Italy) for 20 h. Finally, the obtained films were left at room temperature and humidity for 2 h and then placed in heat-sealed polyethylene bags until they were used for further characterization.

##### Appearance and Folding Endurance

The hydrophilic thin layered films were subjected to visual inspection in order to evaluate their transparency, uniformity and absence of air bubbles. Moreover, to assess their flexibility, folding endurance was evaluated as described in the literature [14]. Briefly, each film was repeatedly folded until it breaks or folded up to 300 times (endpoint) without breaking. The number of folding allowed for each film was reported as the folding endurance value.

##### Preparation of Microcomposite Buccal Thin Films

To prepare SLM-loaded buccal films, 380 mg of previously prepared SLMs were gently mixed with 5 g of gel (C or D matrices), and the homogeneous and bubble-free mixture was dried as previously described. The so-formed films were left to equilibrate at room temperature and humidity for 2 h, then cut into disks by a biopsy punch. The samples were put in polystyrene disposable weighing boats, packed into polyethylene heat-sealed bags and stored at 8 °C to maintain the integrity of SLMs and the flexibility of the film.

##### Uniformity Tests: Weight and Drug Content

To evaluate buccal films uniformity, small film disks having an area of 0.5 cm^2^ were carefully obtained by using a biopsy punch. Three randomly selected disks from each batch were accurately weighed and then employed to evaluate drug content uniformity. Each film disk was completely dissolved into a distilled water/methanol mixture (1:4 *v*/*v*) and then brought to volume (5 mL) with a fresh solvents mixture. The amount of MCZ was determined by UV-VIS measurements (Shimadzu 1700 Instrument, Japan) at λ = 272 nm using the relative blank and calibration curve (see above). The same procedure was performed on microcomposite buccal films loaded with empty SLMs in order to avoid any interference of the excipients employed. The DL% (drug loading percentage) was calculated as the amount of MCZ (mg) encapsulated into 100 mg of buccal film. The LE% (loading efficacy percentage) was calculated as the amount of MCZ (mg) actually encapsulated relating to the total amount of MCZ (mg) contained in the starting amount of SLMs used. The uniformity evaluations were performed on three batches and reported as mean ± SE (*n* = 9).

##### Swelling Studies

The extent of swelling was determined in triplicate on portions (1 cm^2^) of each SLMs-loaded buccal film by using pH 6.8 simulated salivary fluid pre-warmed at 37 ± 0.5 °C as the soaking fluid. The samples were carefully weighed (Wd) and incubated at 37 ± 0.5 °C in simulated salivary fluid pH 6.8 (1 mL). At definite time intervals (5, 10 and 15 min), the exceeding fluid was gently removed by a filter paper. The swollen disk was then reweighed (Ws), and the swelling degree was calculated using the following equation:

Swelling Degree (SwD) = (Ws − Wd)/Wd

where Ws is the weight of the swollen sample after time t and Wd is the initial weight. Results are expressed as the mean ± SE of six experiments [15].

##### Ex Vivo Mucoadhesion Strength Measurement

The ex vivo mucoadhesive strength evaluation of MCZ loaded films was performed by the modified two-armed physical balance method [16]. The inner part of the porcine cheek excised from just-slaughtered pigs was used as model tissue and handled without any pre-treatment. The tissue was glued by cyanoacrylate resin (Super Attak Loctite^®^, Henkel Italia Srl, Milan, Italy) on a glass support (Petri dish) and placed in a thermostatic bath at 37 ± 1 °C, which was maintained throughout the experiment. The film’s disk (area 0.5 cm^2^) was fixed using a bi-adhesive to the lower side of a rubber stopper hanging from the balance arm. Before starting the measurements, the porcine tissue was wetted with 50 µL of simulated salivary fluid, and then the film was placed on the tissue so it just touched the mucosal surface, and a light force with a fingertip was applied for 20 s. Then, the measurements started 3 min after application. Two disks, taken from three different films, were used to perform the test, and the results were expressed as mean ± SE (*n* = 6).

The grams required to detach the film from the mucosal surface provided the measurement of mucoadhesive strength, according to the equation:

Force of adhesion (N) = (g × 9.81)/1000

Then, the detachment force was calculated as follows:

Detachment force (N/m^2^) = Force of adhesion (N)/Surface area (m^2^)

#### 2.2.11. Ex Vivo Evaluations

##### Tissue Preparation

Mucosal specimens, consisting of tissue removed from the vestibular area of the retromolar trigone (buccal mucosa) of freshly slaughtered domestic 6–8-month-old pigs (intended for human consumption), were collected and immediately transferred to the laboratory in a refrigerated transport box within 1 h of animal sacrifice. Excesses of connective and adipose tissue were trimmed away, and then specimens were placed in PBS solution containing trehalose (5% *w*/*v*) as the cryoprotectant, left for 1 h and kept at −80 °C for at least one week. Before the ex vivo permeation studies, the specimens were washed for 1 h in PBS and then subjected to thermal shock in order to obtain the buccal mucosa. Briefly, tissue samples were dipped for approximately 2 min in a pre-warmed isotonic solution (60 °C), and then the connective tissue was carefully peeled off from the mucosa (250 ± 25 mm thick) to obtain the heat-separated epithelium along with the intact basal lamina [17]. The thickness was measured using a digital micrometer (VWR International, Milano, Italy).

##### Ex Vivo Permeation Studies

The obtained buccal mucosa was equilibrated in PBS solution for about 1 h at room temperature to remove biological matter that could interfere with drug analysis [18]. Afterwards, appropriate sections of mucosa were mounted in vertical jacketed, Franz type diffusion cells (Permeagear, flat flange joint, 9-mm orifice diameter, 15 mL acceptor volume, SES GmbH—Analysesysteme, Bechenheim, Germany), used as a two-compartment open model. The mucosal tissues were equilibrated at 37 ± 0.5 °C by adding citric acid solution with a pH of 3 (chosen in order to maximize MCZ solubility) in both the donor and the acceptor compartments. Hereafter, the citric acid solution was removed from the donor compartment and replaced with the donor samples. At scheduled time intervals (60 min), samples (0.5 mL) were withdrawn from the acceptor compartment and immediately replaced with a fresh citric acid solution in order to maintain sink conditions. Each experiment was carried out at 37.0 ± 0.5 °C, for 6 h and repeated six times. The donor samples analyzed were the following: 80 mg of Daktarin^®^ gel 2% *w*/*w* (containing 1.6 mg of MCZ) employed as commercial dosage to form the reference; 15 mg of SLMs (containing 1.2 mg of MCZ); 2.5 mg of SLMs (containing 0.2 mg of MCZ); 22.5 mg of SLM-loaded gel (containing 0.2 mg of MCZ); 0.5 cm^2^ of buccal film disk D (containing 0.86 mg of MCZ). Results were reported as means ± SE. The amount of MCZ permeated through the porcine buccal mucosa was determined by spectrophotometric measurements at λ = 272 nm. MCZ standard solutions in citric acid 1 mM (pH 3) were used to construct the calibration curve (concentration range: 0.02–0.50 mg/mL; y = 1.386x + 0.004; R = 0.999).

##### Evaluation of MCZ Amount Entrapped into the Buccal Tissue

At the end of each ex vivo permeation experiment, the amount of MCZ entrapped in the mucosal tissue was extracted and quantified. Each mucosal specimen was rapidly washed with 5 mL of isotonic solution and then dipped in methanol (5 mL), subjected to sonication (Branson B1200, Branson Ultrasonic Corporation, Danbury, CT, USA) for 5 min and maintained in the extraction solvent at room temperature for 24 h. After that, the extraction liquor was collected, brought to volume with fresh methanol and the amount of drug extracted was quantified by UV-VIS analysis. Moreover, the same procedure was carried out on mucosal specimens treated with 15 mg of SLMs (containing 1.2 mg of MCZ) whose experiments were interrupted at different time points from 1 to 6 h in order to evaluate the time required to saturate the tissue with the drug.

#### 2.2.12. Data Analysis

Data were expressed as mean ± SE. All differences were statistically evaluated by the Student’s *t*-test with the minimum levels of significance as *p* < 0.05.

## 3. Results and Discussion

### 3.1. Synthesis and Characterization of Cetyl Decanoate

**Figure 1:**
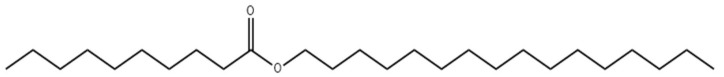
Chemical structure of cetyl decanoate.

Cetyl decanoate (synonyms: hexadecyl decanoate, cetyl caprate, decanoic acid hexadecyl ester; CAS 29710-34-7) is the ester between capric acid and cetyl alcohol (Figure 1; C_26_H_52_O_2_; MW 396.7 g/mol). It is an aliphatic ester without unsaturation and, therefore, very stable. Moreover, it shows high solubilizing power against lipophilic molecules and exhibits remarkable “skin feel” properties, probably due to its structural similarity with the lipid portions of the epidermis and mucosal epithelia [19]. It is widely used as a mixture under the INCI name of “cetyl esters” and as a component of mixtures of esters obtained from the processing of vegetable oils (see Cocoyl Caprylocaprate European Pharmacopoeia monograph, Ph. Eur.) both in the cosmetic and pharmaceutical fields as an emollient and skin conditioner. However, nowadays, nothing has been reported about its use as a standardized raw material aimed at conferring ad hoc properties to the obtained product, nor its use for the preparation of SLMs. Additionally, several methods have already been reported to synthesize cetyl decanoate [20,21,22,23], but the here proposed synthetic approach is novel and based on the reaction of decanoic acid chloride and cetyl alcohol without the use of metal catalysts.

**Figure 2:**
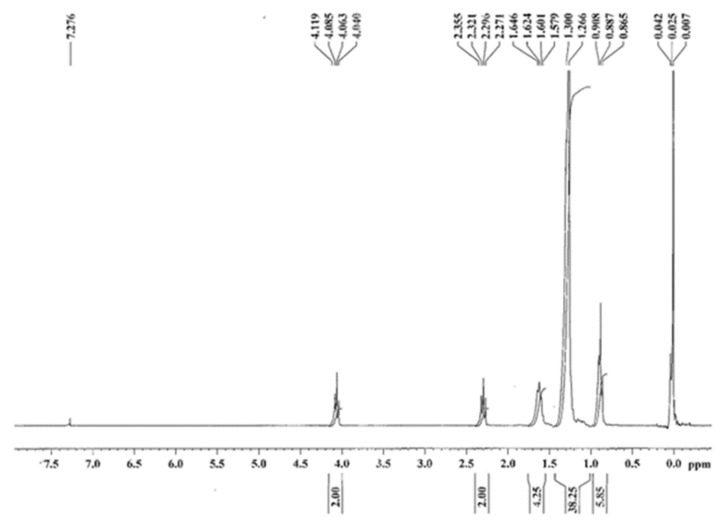
^1^H-NMR of cetyl decanoate.

**Figure 3:**
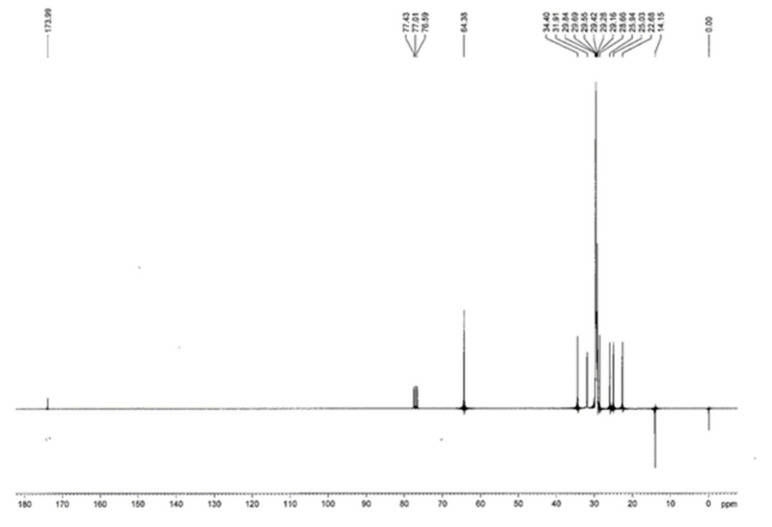
^13^C-NMR of cetyl decanoate.

**Figure 4:**
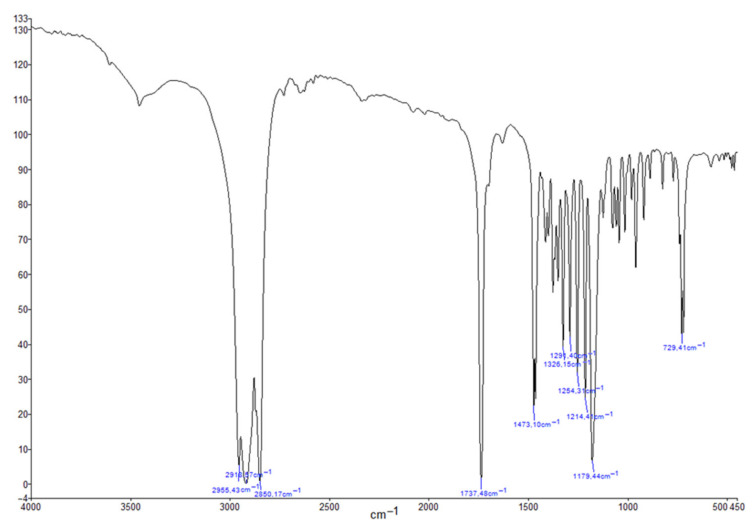
FT-IR of cetyl decanoate.

The obtained purified product was subjected to several spectroscopic evaluations in order to demonstrate the success of the synthetic strategy and the actual complete purification from the starting unreacted products. All the ^1^H-NMR (Figure 2), ^13^C-NMR (Figure 3) and FTIR (Figure 4) (enlarged versions of Figure 2, Figure 3 and Figure 4 are available as Supplementary Materials) data confirm the obtainment of the desired product and its purity. Data were further confirmed by the melting point evaluation, which resulted in 32 °C.

### 3.2. Optimization and Characterization of MCZ-Loaded SLMs

Solid lipid microparticles (SLMs) are lipid-based carriers, which derive from conventional oil-in-water (o/w) emulsions by replacing the liquid oil phase with a solid (at room temperature) lipid component or lipid mix [13]. Due to their composition, SLMs could be excellent carriers for lipophilic drugs. To design SLMs, the choice of the lipid excipients to be used is crucial as they will affect the properties of the final drug delivery system in terms of both shape, dimension, homogeneity and drug loading capacity. At this point, one of the key factors affecting drug loading ability and drug release kinetics is the lipid’s miscibility in the melted status [11,12]. In this work, 1-hexadecanol and cetyl decanoate have been selected as lipid mixture matrices. 1-hexadecanol was chosen as the long-chain aliphatic alcohol having good miscibility with aliphatic esters and, at the same time, self-emulsifying properties [24]. Moreover, 1-hexadecanol is considered safe and, consequently, is widely used in dermatological products.

As 1-hexadecanol and cetyl decanoate amounts are critical in determining the SLMs characteristics, various ratios were tested, ranging from 10:90 to 90:10 *w*/*w*.

Miconazole (MCZ), a potent, lipophilic, antifungal molecule, was chosen as the lipophilic drug model to be conveyed by SLMs. MCZ was homogeneously embedded into the lipid matrix in order to obtain SLMs with the aims of promoting drug entry and accumulation into the epithelial tissues and potentially increase its efficacy for the topical treatment of OC.

In order to maximize the drug content in the SLMs, all attempts to microencapsulation have been performed considering the contribution of 10% in MCZ with respect to the total mass of the drug and lipids. The best results were obtained when combining MCZ:1-hexadecanol:cetyl decanoate in the ratio 10:72:18 *w*/*w*.

To prepare SLMs, several methods have been reported in the literature: hot-melt microencapsulation technique, melt emulsification method, solvent emulsification-evaporation method, solvent emulsification-diffusion method, double emulsion (w/o/w) method, sonication method, spray congealing method, supercritical fluid-based method and spray drying method [25,26,27,28,29]. In this work, the hot-melt microencapsulation technique was used. The lipophilic drug was dissolved into the melted lipid mix, and the hot clear mixture is emulsified into a pre-warmed water solution. Afterward, the obtained o/w emulsion was poured into an ice bath in order to promote quick solidification of the lipid phase, thus obtaining the SLMs [11,13].

The key factors affecting the suitability of the preparation method were the following: the pH of the employed aqueous medium and the rate during the emulsification process.

Generally, MCZ has been successfully entrapped because of its poor aqueous solubility and hence low partitioning into the external aqueous phase during microsphere formation. However, the entrapment amount of MCZ was pH-dependent. As the pH value of the external phase increased, the solubility of MCZ lowered, thus increasing the encapsulated amount of the drug. The maximum DL% value (8 ± 0.5%) was obtained at pH 8.0 (due to Na_2_CO_3_). The decrease of the pH value to 7.0 dropped the SLMs drug content below 6%.

The addition of a surfactant was not necessary to obtain spherical particles, probably due to the emulsifying activity of 1-hexadecanol. Furthermore, the insertion of limonene in the aqueous phase, though being immiscible with it, led to the encapsulation of very small amounts of terpene in SLMs, proven by the strong smell emanated by the microparticles when crushed between the fingers. The presence of limonene in SLMs could promote MCZ absorption into mucosal tissues due to its well-known properties as a chemical penetration enhancer [30].

The main other factor affecting the size distribution was the stirring rate used during the SLMs preparation. A restricted range of dimensional distribution and a high yield percentage value (97.0 ± 0.5% *w*/*w*) related to the starting material were obtained at a stirring speed of 800 rpm. All batches produced in such a way resulted as reproducible in terms of particle sizes, indicating that stirring condition, cooling rate and separation process were well controlled. The batches produced by increasing the stirring rate (800–1200 rpm) resulted in a sharp decrease of the average particle size, as well as in the particle recovery yield. Recovery was lower when the particle size was smaller because smaller SLMs could be lost during the filtration process. Accordingly, larger particles were obtained by setting the stirring speed to values less than 800 rpm. However, in these cases, a reduction of the recovery yield was also observed due to lipid material adhesion to the beaker walls during the cooling process.

The obtained optimized MCZ-loaded SLMs were subjected to further characterization in order to evaluate the efficacy of drug encapsulation, as well as their homogeneity in terms of dimension and shape.

To determine the MCZ content, UV-VIS analysis has been performed. The drug loading % (DL%) resulted in 8.0 ± 0.5% (*w*/*w*) and thus the loading efficacy % (LE%) value was 80 ± 5%. These data confirmed the suitability of the microencapsulation technique, even if a little but acceptable loss in MCZ was observed. The presence of MCZ into the microsphere was also confirmed by the FTIR analysis of MCZ loaded SLMs versus MCZ crystalline pure (Figure S1).

**Figure 5:**
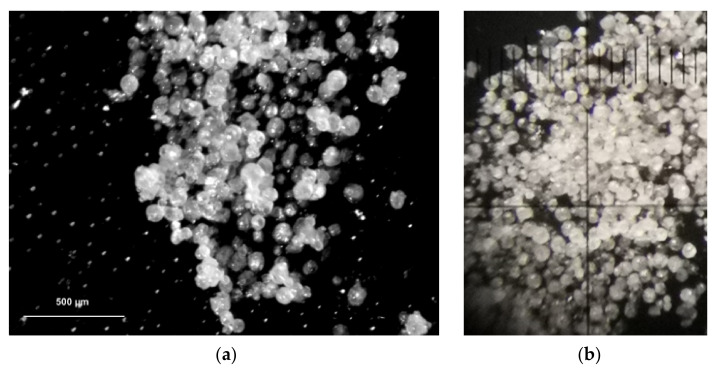
SLMs morphological analysis: photographs of some representative samples collected by Leica M165C (Leica Microsystems, Germany) optical microscope: (**a**) equipped with a Photometrics digital camera DFC450 and Leica application Suite LAS4 software (40× magnification); (**b**) manually by an external camera.

To evaluate SLMs shape and dimension, morphological analysis has been conducted by optical microscopy (Figure 5).

As observable, the collected photographs confirmed the presence of spherical microparticles, approximately in the range of 100–200 µm. Moreover, the absence of irregularly shaped particles or crystalline material between the SLMs is evident, suggesting that no crystallization phenomena of MCZ occurred during the cooling step and the formation of the SLMs by the starting hot emulsion. To evaluate the dimensional distribution of the microspheres, two methods were used: sieves and light scattering analyses. The first provided information on the size distribution of the coarse particles and simultaneously enables the separation of the particles according to classes, highlighting the potential presence of non-spherical aggregates of lipid material (Table 2). The second method provided information on small particles that cannot be measured by sieving (see Figure S2 in the Supplementary Materials to observe the particle size distribution graph).

Both sieve and light scattering analyses showed that most of the isolated SLMs were of the particle size range 45–310 µm. Light scattering analysis highlights that about 18% of the microparticles ranged from 3 to 45 µm, whereas by sieve analysis, these resulted in 6.7%. This difference in the data is likely due to the method used. Indeed, during sieving, very small particles adhere to the larger ones, thus not falling into the lower sieve. The light scattering analysis, being in dispersion, was not susceptible to this drawback.

These data indicate that the obtained powder is sufficiently homogeneous to handle and workable (e.g., processing, mixing, to be used as intermediate for semisolid or solid formulations) and that the prepared SLMs are small enough to distribute on the tissues well but not too small to be lost during application or even inhaled during handling.

Two other crucial characteristics to be considered and evaluated are the SLMs melting point as well as the physical state of MCZ (amorphous or crystalline). Both these evaluations could be relevant in determining the usefulness of the proposed microparticles and thus the potential enhanced effectiveness of MCZ when embedded into SLMs.

The SLMs melting point could be a key parameter. The melting points of each component of the SLMs and the MCZ-loaded microparticles as a whole are reported in Table 3.

As observable, the proposed drug-loaded SLMs showed a melting point value compatible with the physiological temperature of the oral cavity. This could lead to SLMs melting when applied strictly in contact with the buccal mucosa and, consequently, the release of MCZ as molecular dispersion. This could lead to an improvement in drug penetration into the mucosal tissue and thus increased effectiveness.

Another relevant parameter to be considered is the physical state of the encapsulated drug. Indeed, the obtainment of the amorphous form is generally preferred in order to promote drug solubility and, as a consequence, absorption/accumulation and efficacy. To evaluate whereas MCZ was amorphous or crystalline when embedded into the SLMs, DSC analyses were conducted.

**Figure 6:**
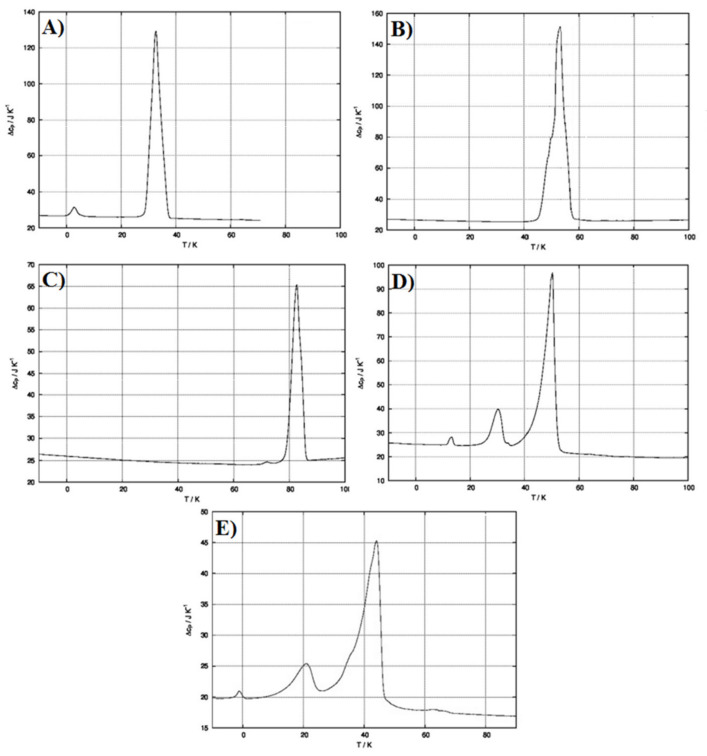
DSC thermograms of: (**A**) pure cetyl decanoate; (**B**) pure 1-hexadecanol; (**C**) pure MCZ; (**D**) physical mix of cetyl decanoate, 1-hexadecanol and MCZ; (**E**) MCZ-loaded SLMs.

Figure 6A–C reports the DSC thermograms of every single component of the SLMs by itself. As observable, each raw material is characterized by the presence of an endothermic peak related to its previously reported melting point. Figure 6D shows the thermogram obtained after physical mixing of all the components in the appropriate and selected ratio. It is possible to highlight that the simple mixing procedure already led to drug-excipient interactions, which allow drug amorphization. Indeed, the two endothermic peaks related to cetyl decanoate and 1-hexadecanol are still present, while the MCZ endothermic peak is missed. This is, obviously, also observable in the SLMs thermogram (Figure 6E). Moreover, it is important to notice that the ratio between the intensity of the two endothermic peaks related to the excipients reflects their actual ratio in terms of microparticles composition (the enlarged version of Figure 6 is reported in the Supplementary Materials).

### 3.3. Preparation of SLMs-Loaded Buccal Gel and SLMs-Loaded Buccal Film

As the aim of this work was to propose an effective and innovative therapeutic strategy to replace the currently available ones in the treatment of OC while overcoming their relevant limitations, it is important to conceive and design an easily applicable dosage form having good patient compliance. Although SLMs are promising candidates for improving the efficacy of MCZ by enhancing its penetration into the buccal tissue, they consist of a powder, which could result in difficulty to evenly distribute inside the oral cavity, thus being not patient-friendly, leading to the administration of poorly reproducible doses, thus compromising the therapeutic outcomes.

From this view, the prepared MCZ-loaded SLMs were alternatively embedded into a buccal gel and a buccal film in order to obtain easily administrable, comfortable and homogeneous dosage forms.

To prepare the SLMs-loaded gel, hydroxyethylcellulose (HEC), trehalose and PVP-K90 were chosen as excipients in order to obtain a homogenous, transparent and bubble-free gel in which to incorporate MCZ-loaded SLMs. HEC is a gelling and thickening agent derived from cellulose and widely used in the cosmetic and pharmaceutical fields [31]. Polyvinylpyrrolidone (PVP) is a biocompatible, biodegradable, water-soluble polymer often used to prepare formulations with strong mucoadhesive properties [32,33]. Trehalose (α-D-glucopyranosyl-α-D-glucopyranoside) is a nonreducing sugar widely used in food, cosmetics and pharmaceutics for its unique ability to sustain and preserve a wide array of biological molecules due to its functions as a stress protectant, preventing protein degradation and preserving the cell membrane structure under stress conditions [34]. In addition, new findings suggest that the targeting of the trehalose pathway might compromise fungal viability and virulence in *Cryptococcus neoformans* [35], indicating this sugar as a potential antifungal agent [36]. Finally, trehalose possesses sweetening properties suitable for diabetic patients, which could improve the palatability of the formulation. To enhance palatability and prevent microbial proliferation, thus extending the expiration date of the formulation, limonene was added to formulations B and D. Moreover, as already reported, limonene is well-known as a chemical penetration enhancer, and thus, it could further improve the effectiveness of the proposed formulation.

MCZ-loaded SLMs were added to the obtained optimized gel (ratio SLMs:gel = 1:8 *w*/*w*). The obtained SLMs-loaded gel (appearance was shown in Figure S3, see Supplementary Materials) was evaluated in terms of drug distribution homogeneity in the semisolid mass, resulting in 0.89 ± 0.04% in terms MCZ.

To prepare the SLMs-loaded buccal film (also indicated as microcomposite), firstly, a screening intended to identify the best formulations was performed. Four different film compositions were tested starting from HEC and PVP-K90 as principal excipients and varying in terms of presence/absence of trehalose and limonene as further components. The first formulation screening step consisted of the preparation of empty buccal films (Table 1, see Section 2) to be evaluated in terms of appearance and folding endurance. Film A and B were immediately discharged as they were rigid and brittle when subjected to the folding endurance evaluation. On the other hand, films C and D did not show any cracks even after folding more than 300 times. Furthermore, they were transparent, air bubble-free and flexible. This evidence is probably due to the presence of trehalose, which has a high water retention capacity and thus acts as a plasticizer, allowing suitable films with the desired characteristics to be obtained. In view of these encouraging results, two SLMs-loaded buccal films (according to C and D films compositions) have been prepared by the solvent casting method.

The proposed film preparation method is simple, inexpensive and does not imply the use of organic solvents. The method was the most appropriate to obtain microcomposite formulations characterized by a homogenous dispersion of SLMs embedded into the hydrophilic solid matrix system resulting in highly reproducible products. The oven temperature to dry the prepared gel, thus obtaining the final microcomposite, was fixed at 30 °C to avoid SLMs melting, whereas to establish the drying time, preliminary tests were conducted. The best results were obtained after 20 h of drying in an oven. A drying procedure protracted over 28 h leads to brittle and stiff films.

**Figure 7:**
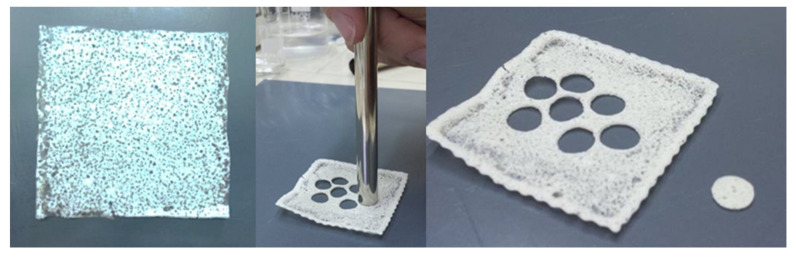
Appearance of the SLMs-loaded buccal films and preparation of the small disks.

From the obtained formulations, small disks (area 0.5 cm^2^) were collected by the careful use of a biopsy punch (Figure 7) and subsequently used for further studies.

The reproducibility of the films was assessed by measuring the average weight and drug content of three disks obtained from the same batch and repeating this procedure on three different batches. The obtained data are reported in Table 4.

All data confirmed high product reproducibility, though the microcomposite film D shows slightly better characteristics.

Another relevant parameter to be considered in the design of an effective buccal formulation is the swelling degree. This parameter is related to the ability of the formulation to absorb water from the surrounding environment and swell, thus influencing film bioadhesion and drug release. Indeed, the amount of water absorbed must be the compromise between the development of the interactions through dosage form and mucosal mucins and the increase in the volume of the formulation, which, if excessive, could cause patient discomfort [37]. Formulations characterized by too high a swelling degree risk being too bulky, causing discomfort for the presence of an extraneous body in the oral cavity. The water uptake of the films was quantified gravimetrically at three different time points (5, 10 and 15 min) by using artificial saliva as the swelling fluid. Data are reported as a swelling degree of the SLMs-loaded buccal films, which resulted in a maximum after 5 min being 7.54 ± 0.91 and 6.70 ± 0.87 for SLMs-loaded film C and SLMs-loaded film D, respectively. After that, no more water was absorbed, and the microcomposite films remained unmodified (Figure S4).

These results indicate that the microcomposite films, despite the presence of a lipid portion, are able to uptake the environmental fluids and swell, producing a dense layer of gel capable of retaining the SLMs on the target site. A seven or eight-fold weight increase determines a variation in volume that should not be uncomfortable. Additionally, the application of the film could protect the OC lesions from further external insults.

While both films were promising in terms of uniformity and swelling degree, SLMs-loaded film D was chosen as the final formulation to be further characterized, as it contains limonene, which could additionally improve patients’ compliance.

The mucoadhesive properties of a buccal thin layer film are fundamental, as long as they influence the ability of the formulation to be retained on the target site avoiding intraoral detachment and the consequent ingestion. The bioadhesive behavior of a dosage form can be studied by using a wide variety of methods, which are influenced by instrumental variables and experiment design [38]. Here, the mucoadhesiveness of the proposed buccal film was measured by using an analytical balance modified according to the literature [16]. Data are expressed as a force of adhesion and detachment force, calculated as reported in the equations described in the Section 2.2. The force of adhesion and the detachment force of SLMs-loaded film D resulted in 0.1223 ± 0.015 N and 2446.43 ± 53.57 (N/m^2^), respectively, confirming suitable mucoadhesive properties.

### 3.4. MCZ Permeation/Penetration Enhancement Studies: Formulations Comparison

The final proof of concept to evaluate the possible usefulness of the proposed formulations, as well as the actual effectiveness of SLMs to act as penetration and permeation enhancers for a lipophilic drug, such as MCZ, consists in the ex vivo evaluation. To better highlight the potential of the here proposed novel dosage forms, all the preparations (SLMs, buccal gel and buccal film) were compared with a currently available, well-known and widely employed formulation: Daktarin^®^ oral gel, containing 2% of MCZ (*w*/*w*).

The ex vivo tests were conducted by using vertical Franz cells and porcine buccal mucosa. However, due to the poor solubility of MCZ in buffer pH 7.4, this cannot be used as a receiving fluid because it will imply the absence of the sink conditions required to observe the permeation phenomenon. To overcome this drawback, 1 mM citric acid solution (pH 3) was used as the acceptor fluid, as it can cause protonation of the imidazole ring (pKa ≈ 6.5) and, therefore, makes MCZ partially water-soluble.

**Figure 8:**
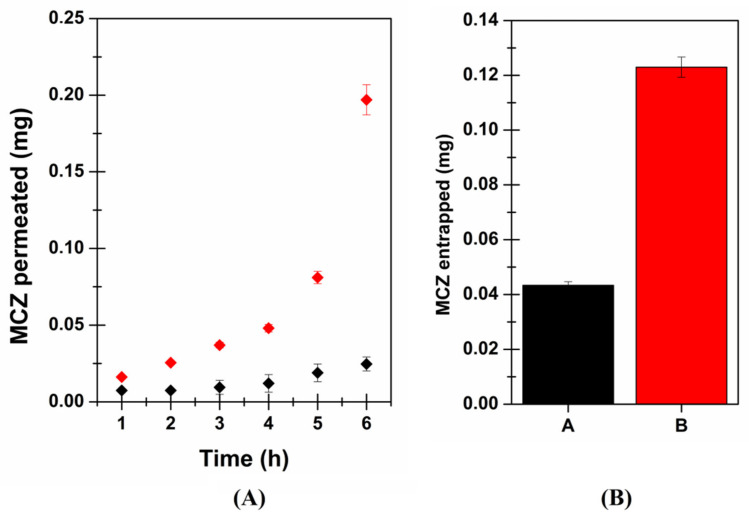
MCZ (mg) after administration of Daktarin^®^ oral gel (corresponding to 1.6 mg MCZ—black) or SLMs (corresponding to 1.2 mf MCZ—red): (**A**) permeated throughout porcine buccal mucosa; (**B**) entrapped into the porcine buccal mucosa after 6 h. Data are presented as mean ± SE (*n* = 6).

Firstly, MCZ’s ability to cross the buccal membrane was evaluated after administration of Daktarin^®^ oral gel (80 mg = 1.6 mg of MCZ) and MCZ loaded SLMs (15 mg = 1.2 mg of MCZ). While the amount of MCZ into the acceptor fluid was quantified at different time points until 6 h, at the end of each experiment, the amount of MCZ entrapped into the buccal tissue was also evaluated. Figure 8 reports the obtained MCZ permeation profile and the amount of MCZ entrapped into the mucosa after six hours of the application of two formulations.

As observable, the encapsulation of MCZ into solid lipid microparticles determines a significant permeation enhancer effect. This is probably due to the ability of the SLMs to melt when kept in contact with the epithelia at 37 °C, fuse with the physiological lipids and penetrate the mucosa entraining the solubilized MCZ. Mucosal entrapment values also indicate that SLMs increase the MCZ capability of partitioning into the mucosal tissue and accumulating.

In particular, the values obtained in terms of MCZ entrapped into the membrane (0.0434 ± 0.0013 and 0.123 ± 0.0037 mg for Daktarin^®^ oral gel and MCZ loaded SLMs, respectively) suggested a high affinity between the SLMs components and the lipids of the epithelium, thus probably producing the “lipidization” of the tissue, which favors MCZ partition resulting in enhanced drug ability to cross the buccal mucosa due to a high concentration gradient between the mucosa and the acceptor fluid.

To better understand the timing of this effect determined by the SLMs as well as to establish if the membrane reach a saturation condition, a set of permeation experiments was performed to evaluate the amount of MCZ entrapped into the buccal mucosa at different time points (from 1 to 6 h).

**Figure 9:**
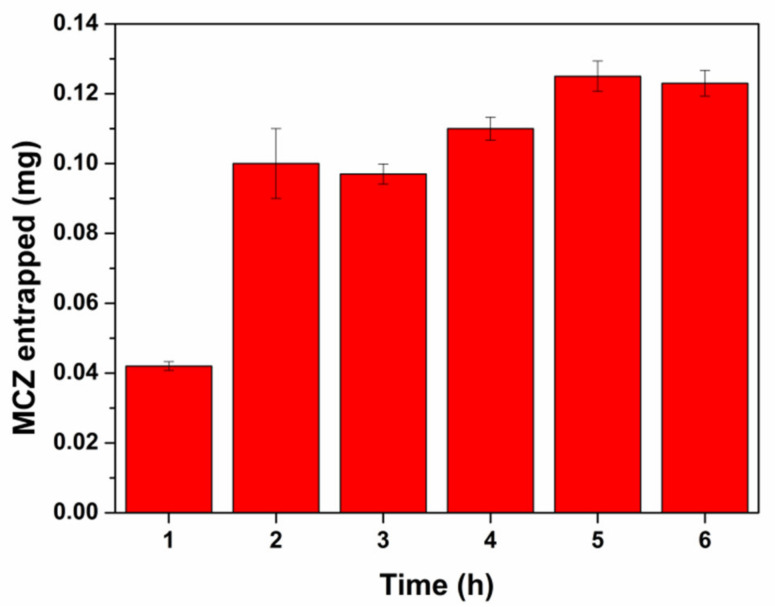
Amount of MCZ (mg) entrapped into the buccal mucosa at different time points after the administration of 15 mg SLMs (corresponding to 1.2 mf MCZ). Data are presented as mean ± SE (*n* = 6).

As reported in Figure 9, the amount of drug entrapped into the tissue after the administration of 15 mg of SLMs (1.2 mg of MCZ) grows with time until a plateau was reached at 5 h (membrane saturation). The maximum amount of MCZ accumulated was 125 µg (10.4% of the dose).

However, the aim of a topical antifungal treatment for the OC is not to promote drug permeation in systemic circulation but to ensure an optimal therapeutic concentration in the target tissue by administering the dose sufficient to reach it. Indeed, plasma levels of the topically administered drug could cause adverse effects [39].

**Figure 10:**
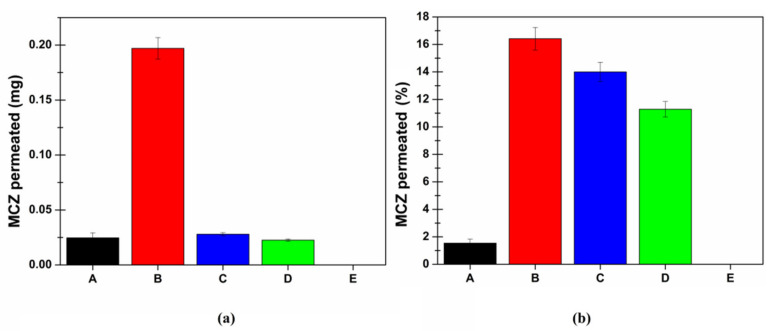
Cumulative amount of MCZ permeated through the porcine buccal mucosa after 6 h of administration, expressed as (**a**) amount (mg) and (**b**) percent of dose for: Daktarin^®^ gel (corresponding to 1.6 mg MCZ—black A); 15 mg of SLMs (corresponding to 1.2 mg MCZ—red B); 2.5 mg of SLMs (corresponding to 0.2 mg MCZ—blue C); 22.5 mg of SLMs-loaded buccal gel (corresponding to 0.2 mg MCZ—green D) and 12.3 mg of SLMs-loaded buccal film (corresponding to 0.86 mg MCZ—yellow E). Data are reported as mean ± SE (*n* = 6).

As the results obtained suggest the high permeation enhancing effect of SLMs, it should be convenient to reduce the administered dose. In this view, further ex vivo permeation studies were conducted by loading into the donor chamber 2.5 mg of SLMs (corresponding to 0.2 mg of MCZ). At the same time, the behaviors of the two proposed new formulations, the SLMs-loaded buccal gel and the microcomposite buccal film, administered in comparable doses, were analyzed. In particular, the donor compartment of the Franz cell diffusion was filled with 22.5 mg of SLMs-loaded buccal gel (corresponding to 0.2 mg of MCZ) or 12.3 mg of SLMs-loaded film disk D (corresponding to 0.86 mg of MCZ, 8 mm diameter according to Franz cell orifice). Figure 10 shows the cumulative amount of MCZ permeated after 6 h of experiment both in terms of quantity (mg) and percent of the administered dose.

As observable, the cumulative amount of MCZ (mg) in the acceptor chamber at the end of the experiments (6 h) is approximatively the same when administering Daktarin^®^ gel, 2.5 mg of SLMs or the SLMs-loaded buccal gel. Although, it should be considered that in the case of Daktarin^®^, the administered dose is eight-fold higher than that contained in 2.5 mg of SLMs or in SLMs-loaded buccal gel (1.6 vs. 0.2 mg). Moreover, when administering the SLMs-loaded buccal film, despite the MCZ dose was 0.86 mg, no drug was found in the acceptor compartment after six hours, highlighting that negligible permeation phenomena occur.

**Figure 11:**
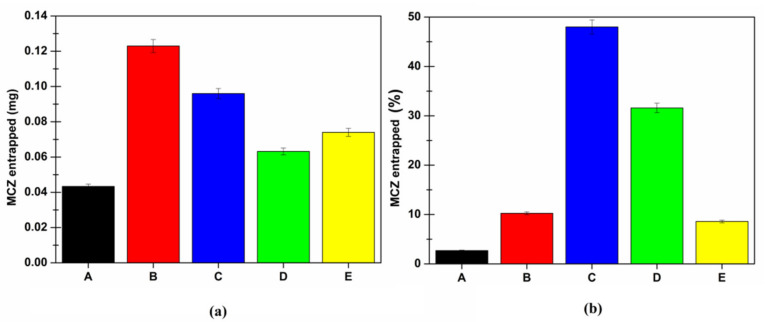
Cumulative amount of MCZ entrapped into the porcine buccal mucosa after 6 h of administration expressed as (**a**) amount (mg) and (**b**) percentage of the dose for: Daktarin^®^ gel (corresponding to 1.6 mg MCZ—black A); 15 mg of SLMs (corresponding to 1.2 mg MCZ—red B); 2.5 mg of SLMs (corresponding to 0.2 mg MCZ—blue C); 22.5 mg of SLMs-loaded buccal gel (corresponding to 0.2 mg MCZ—green D) and 12.3 mg of SLMs-loaded buccal film (corresponding to 0.86 mg MCZ—yellow E). Data are reported as mean ± SE (*n* = 6).

Once again, at the end of the permeation experiments, the amount of MCZ entrapped into the buccal tissue was quantified. Indeed, as already mentioned, the goal of an effective topic drug delivery system to be employed in the treatment of OC is to enhance drug accumulation into the target tissue in order to promote in situ drug effectiveness and, consequently, allow a reduction of the dose to be administered to achieve the therapeutic effect. In Figure 11, the cumulative amount of MCZ entrapped in the buccal tissue after 6 h of application is reported both as the amount (mg) (panel a) and percentage of the dose (panel b).

These findings highlighted the penetration enhancer effect of the SLMs. The amount (mg) of MCZ entrapped in the buccal mucosa was always higher when it was delivered by the SLMs, compared to Daktarin^®^ gel. This effect is more evident when data are expressed as a percentage of the administered dose. It is possible to make a quick and easy comparison between SLMs (dose 2.5 mg, blue bars) and SLMs-loaded gel (green bars) as they are characterized by the same MCZ starting amount (0.2 mg). It is likely to notice that the presence of the buccal gel slightly limits the amount of MCZ entrapped in the mucosa, as well as the amount of MCZ permeated (Figure 11). This is certainly due to the hydrophilic swellable surrounding matrix, which should be crossed by the lipophilic SLMs in order to get in touch with the tissue, melt and promote MCZ accumulation.

Similar behavior is also observed for the SLMs-loaded film, considering the higher dose of MCZ administered. However, it should be noted that the MCZ accumulated in the membrane is consistent, and importantly, it represents approximately 10% of the administered dose. This means that the film is able to release the embedded drug for an extended time, and this could lead to a reduction in the number of daily administrations required.

In any case, the buccal microcomposite film is better than the conventional dosage form, both in the ability to enhance MCZ accumulation in the mucosal tissue and the capability to avoid MCZ bloodstream distribution, which could be potentially unsafe.

Finally, as already mentioned, it is unlikely to propose the administration of free SLMs, and thus, a comparison between the two proposed final formulations (semisolid and solid) must be done. Both of the SLMs-loaded formulations are extremely promising as they are capable of promoting MCZ entry and accumulation into the buccal mucosa, allowing a reduction of the administered dose to achieve the therapeutic effect. Although the results obtained by applying the gel may seem better than those obtained by the film, the advantages of the solid dosage form should be considered. Indeed, a solid dosage form is more stable, easily administrable and patient-friendly compared to a semisolid one. Moreover, the proposed buccal film highlighted no drug permeation, and thus, no MCZ should be lost in the systemic circulation, and no systemic side effects should occur. Finally, a semisolid dosage form could be partially lost by swallowing (thus, consequently, allowing drug loss), remaining, generally, in situ for a shorter period of time than the solid one. This is particularly true when further considering that the proposed buccal film was accurately designed to be mucoadhesive.

## 4. Conclusions

The here reported studies confirm all the starting aims of the present work. Firstly, cetyl decanoate was efficiently synthesized without the use of metal catalysis in order to obtain a pure raw material to be used to prepare MCZ-loaded SLMs. Furthermore, suitable SLMs in terms of DL%, particle size and shape were produced by the hot-melt technique after a careful modulation of some crucial parameters (e.g., pH of the aqueous medium and speed rate during the emulsification process). The prepared MCZ-loaded SLMs also emerged as promising oromucosal penetration enhancers due to their melting temperature range (compatible with the physiologic oromucosal temperature), also thanks to their ability to lead to complete MCZ amorphization. Additionally, SLMs were effectively incorporated into semisolid and solid formulations, which could result in easy administration and being patient-friendly. The ex vivo findings of the present work highlighted that the SLMs act as suitable penetration enhancers in any case. Indeed, the amount (mg) of MCZ entrapped into the buccal mucosa is always higher when it is delivered by the SLMs compared to Daktarin^®^ gel, employed as a model of the conventional dosage form. The findings obtained are even more evident when data are expressed as a percentage of the dose administered. Moreover, it is likely to notice that by embedding the proposed SLMs into a solid or semisolid formulation, a slight reduction in terms of MCZ entrapment into the buccal mucosa is observed. This is certainly due to the surrounding hydrophilic swellable matrix, which should be crossed by the lipophilic SLMs to get in touch with the tissue, melt and promote MCZ accumulation. However, it is unlikely to propose the administration of free SLMs, and thus a comparison between the two proposed formulations must be reported.

At first sight, the results obtained by the ex vivo experiments seem to suggest the superiority of the here proposed SLMs-loaded buccal gel. However, some key factors have to be considered. In particular, the SLMs-loaded buccal film allowed no MCZ permeation, thus totally limiting the possibility of adverse side effects due to MCZ entry into the bloodstream. Furthermore, the consistent amount of MCZ entrapped into the mucosa after the administration of the microcomposite film represents approximately 10% of the administered dose. This suggests that the film is able to release the drug for an extended period of time, consequently leading to a reduction in terms of the number of daily administrations.

Moreover, the SLMs-loaded buccal film could generally benefit from the advantages of a solid dosage form (e.g., stability, ease of administration, high patients’ compliance), while also taking the advantages of an innovative DDS (e.g., mucoadhesion, which results in enhanced retention time on the administration surface vs. semisolid dosage forms, which could be partially lost by swallowing).

## 5. Patents

The work reported in this manuscript is part of an Italian patent application n. IT 201900011436 by Viviana De Caro and Libero Italo Giannola.

## Figures and Tables

**Table 1:** Composition of the gels employed to prepare the buccal films.

Component	Matrix-A	Matrix-B	Matrix-C	Matrix-D
PVP-K90	50 mg	50 mg	50 mg	50 mg
HEC	800 mg	800 mg	800 mg	800 mg
Trehalose	-	-	150 mg	150 mg
Limonene	-	750 µL	-	750 µL
Water	30 mL	30 mL	30 mL	30 mL

**Table 2:** Size distribution by sieving expressed as percent ± SE (*n* = 6).

Particle Size Faction (µm)	SLMs in Each Fraction (% ±SE)
>310	0.33 ± 0.01
250–310	10.02 ± 0.31
200–250	22.40 ± 0.68
125–200	48.00 ± 1.24
90–125	4.85 ± 0.15
45–90	7.75 ± 0.22
<45	22.40 ± 0.68

**Table 3:** Melting points of each component of SLM and MCZ-loaded SLMs.

Compound	Melting Point (°C)
Cetyl Decanoate	32 °C
1-hexadecanol	49.3 °C
Miconazole	83–87 °C
MCZ-loaded SLMs	35–37 °C

**Table 4:** Uniformity evaluation of SLMs-loaded buccal films: weight of the obtained small disks and drug content (as a percentage as mg per disk) reported as means ± SE (*n* = 9).

Sample	Weigh of the Small Disk (mg)	DL% (*w*/*w*)	MCZ (mg) per Disk
SLMs-loaded film C	11.7 ± 1.2	7.09 ± 0.94%	0.83 ± 0.11
SLMs-loaded film D	12.3 ± 0.5	6.99 ± 0.41%	0.86 ± 0.05

## Data Availability

The data presented in this study are available on request from the corresponding author.

## Supplementary Materials

The following are available online at https://www.mdpi.com/article/10.3390/pharmaceutics13091361/s1, Figure S1. FT-IR spectra in KBr of MCZ loaded microspheres (red) and MCZ crystalline pure (blue). Figure S2. Dimensional distribution of a batch of MCZ loaded SLMs by Mastersize 3000, Malvern. Figure S3. Appearance of the SLMs-loaded buccal gel. Figure S4. SLMs-loaded film D after 15 min of swelling. Figures S5–S8 are enlarged versions of Figure 2, Figure 3, Figure 4 and Figure 6.
