# Supplementary material for: Solid and Semisolid Innovative Formulations Containing Miconazole-Loaded Solid Lipid Microparticles to Promote Drug Entrapment into the Buccal Mucosa
Source: Pharmaceutics. 2021 Aug 29;13(9):1361. doi: 10.3390/pharmaceutics13091361 (PMC8468017; doi:10.3390/pharmaceutics13091361)
Supplement: Supplementary file 1 [file pharmaceutics-13-01361-s001.zip › pharmaceutics-1323939-supplementary.pdf]

pharmaceutics

MDPI

Supplementary Materials: Solid and Semisolid Innovative Formulations Containing Miconazole-Loaded Solid Lipid Microparticles to Promote Drug Entrapment into the Buccal Mucosa

Viviana De Caro, Libero Italo Giannola and Giulia Di Prima

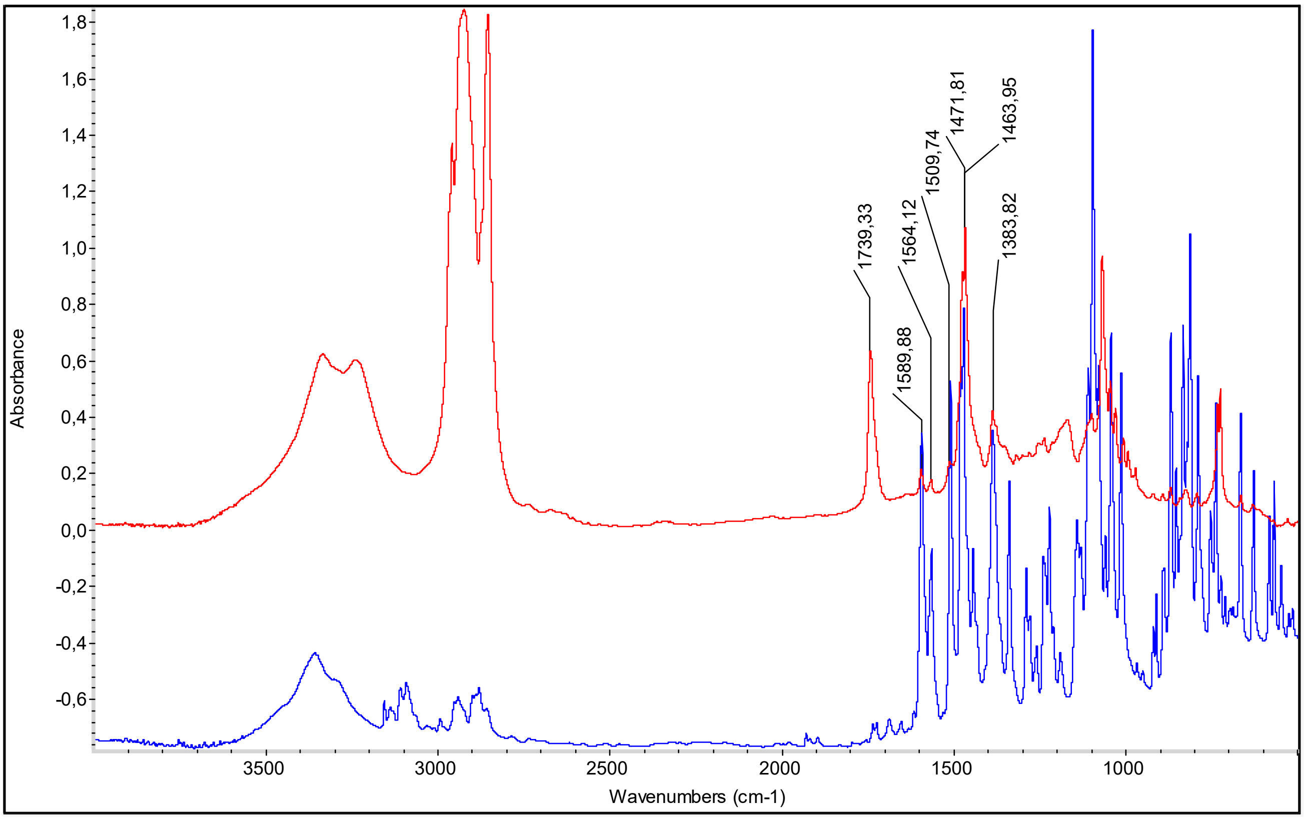

Figure S1. FT-IR spectra in KBr of MCZ loaded microspheres (red) and MCZ crystalline pure (blue).

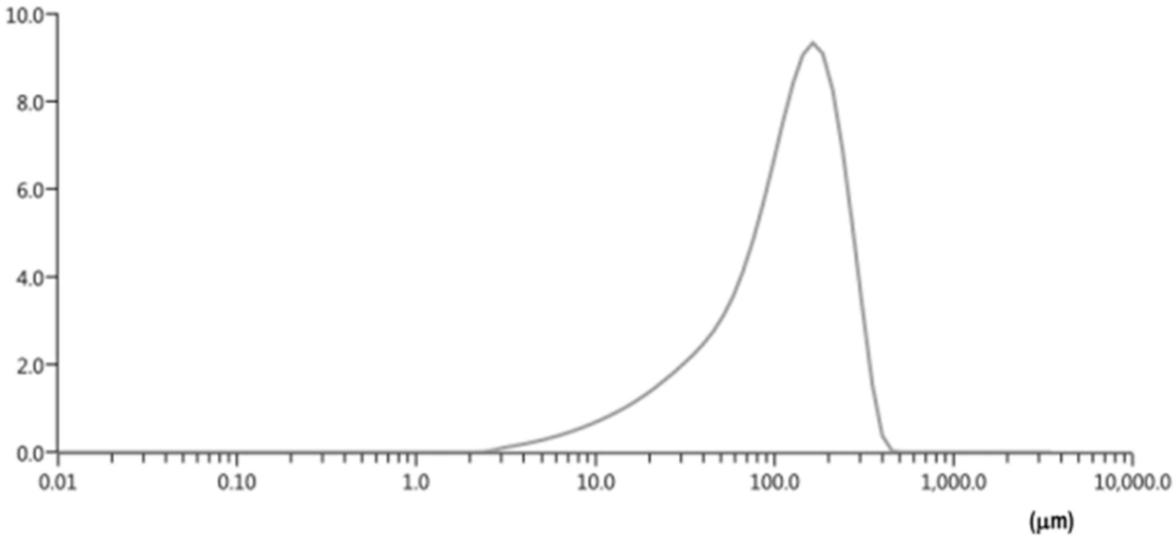

Figure S2. Dimensional distribution of a batch of MCZ loaded SLMs by Mastersize 3000, Malvern.

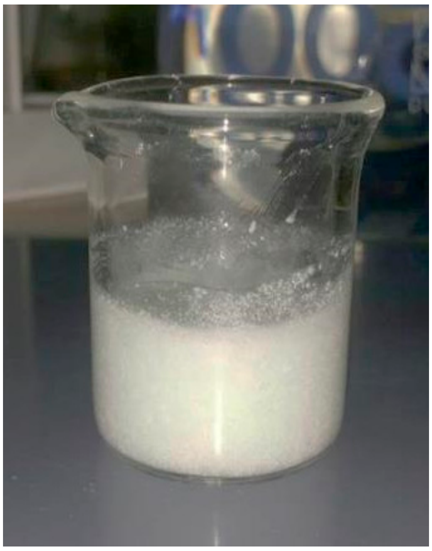

Figure S3. Appearance of the SLMs-loaded buccal gel

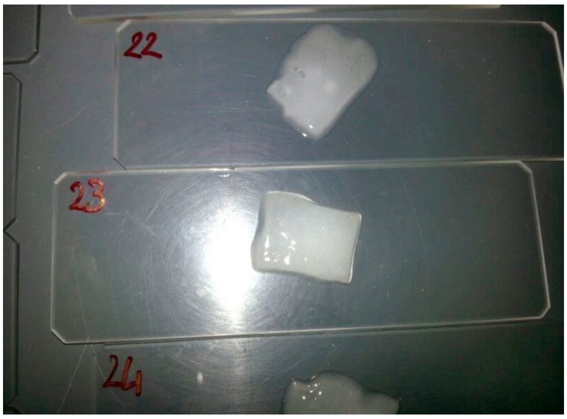

Figure S4. SLMs-loaded film D after 15 min of swelling

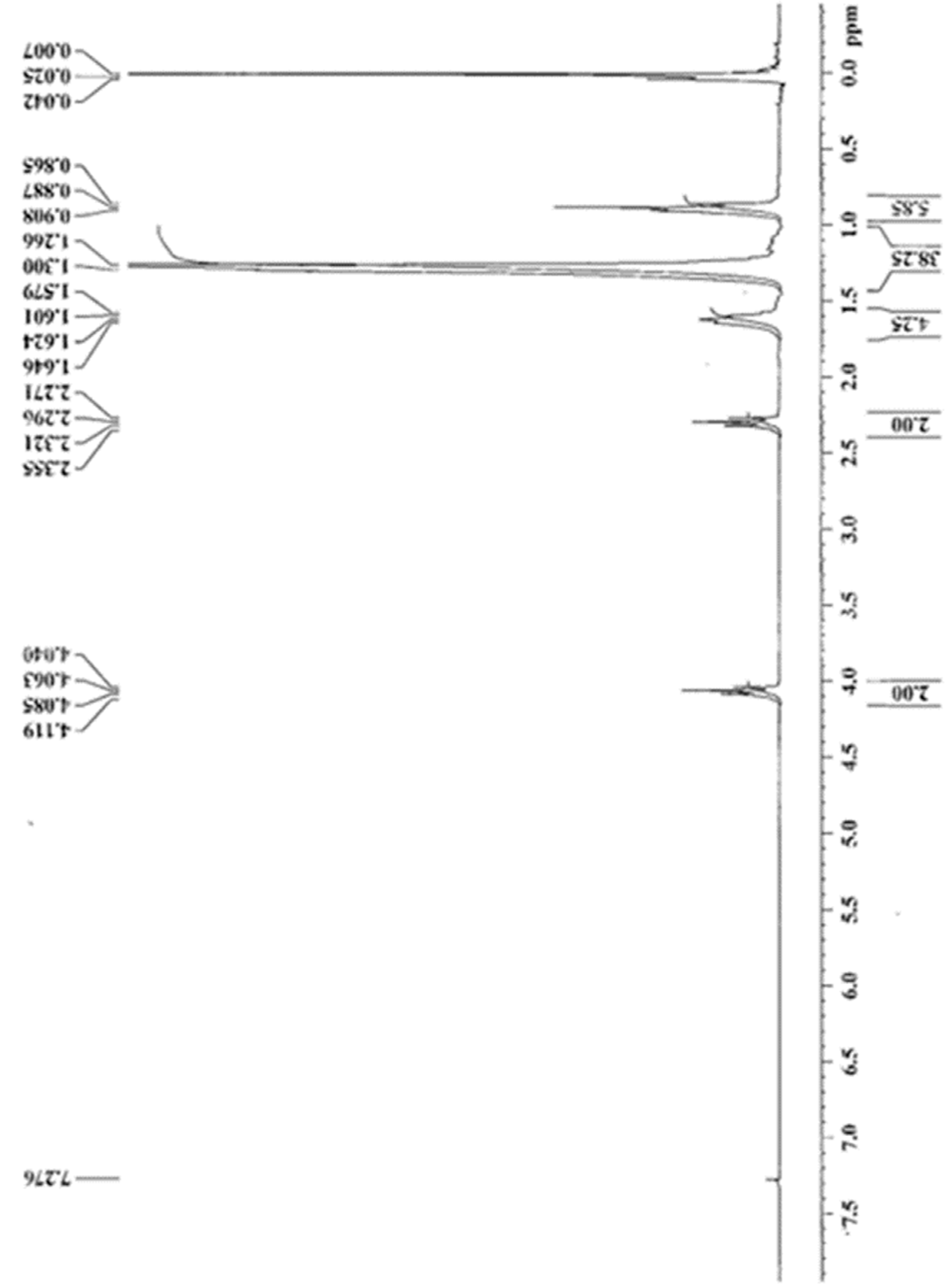

Figure S5. ^{1}H-NMR of cetyl decanoate

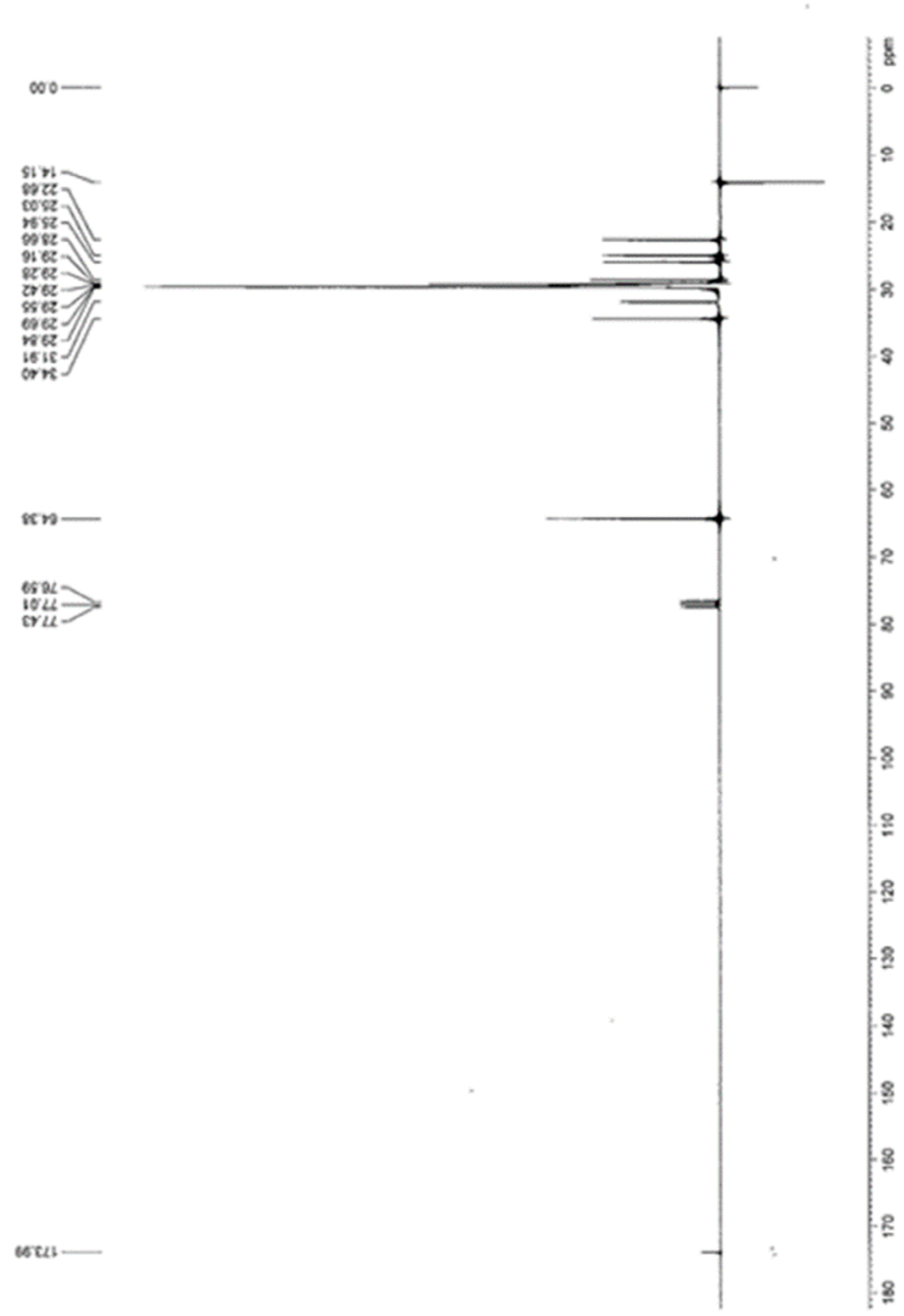

Figure S6. ^{13}C-NMR of cetyl decanoate

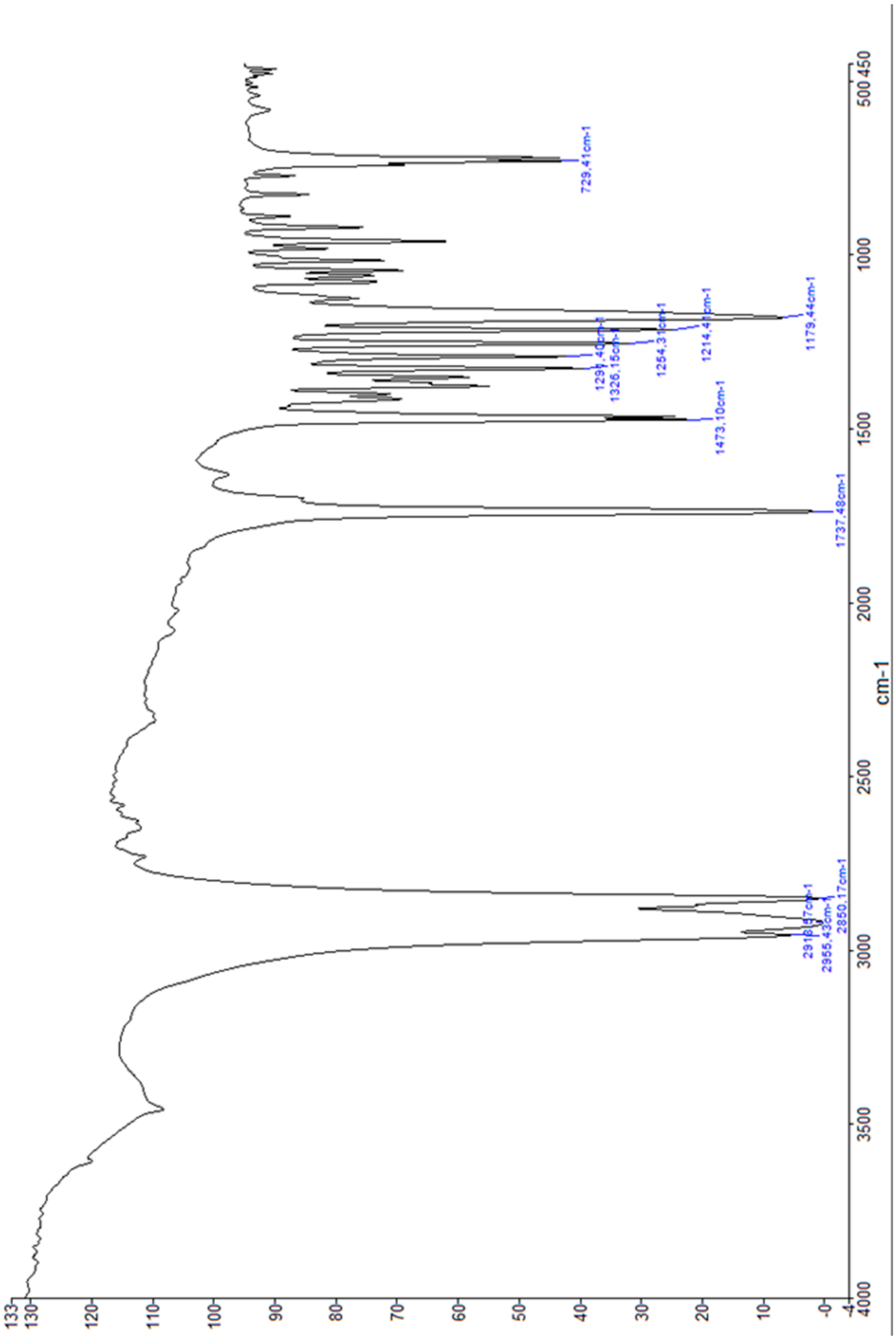

Figure S7. FT-IR of cetyl decanoate

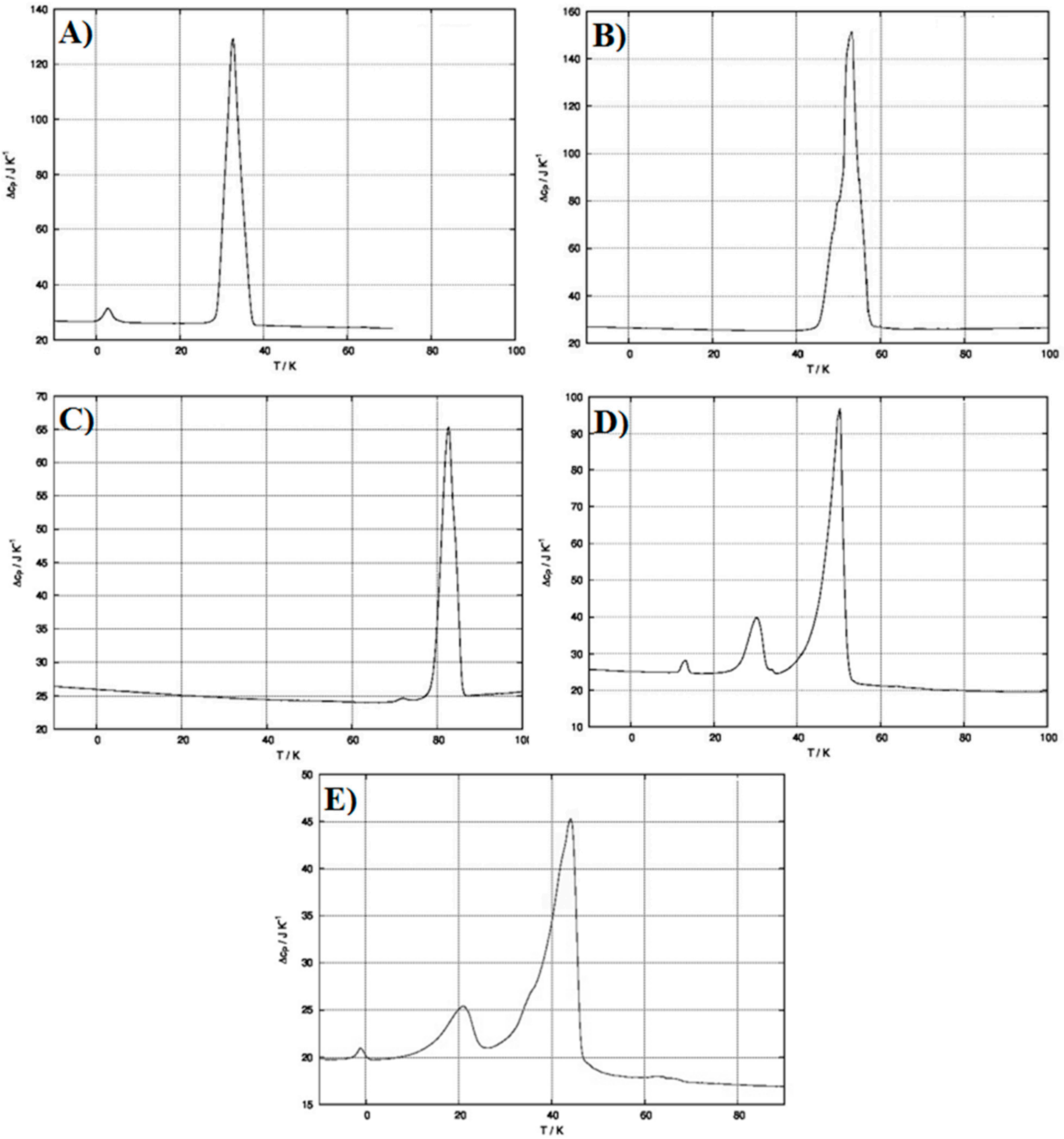

Figure S8. DSC thermograms of: A) pure Cetyl Decanoate; B) pure 1-hezadecanol; C) pure MCZ; D) physical mix of cetyl decanotate, 1-hexadecanol and MCZ; E) MCZ-loaded SLMs
